# CXCL12/SDF-1 in IgG4-Related Disease

**DOI:** 10.3389/fphar.2021.750216

**Published:** 2021-10-26

**Authors:** Riccardo Capecchi, Cristina Croia, Ilaria Puxeddu, Federico Pratesi, Andrea Cacciato, Daniela Campani, Ugo Boggi, Luca Morelli, Antonio Tavoni, Paola Migliorini

**Affiliations:** ^1^ Immuno-Allergology Unit, Department of Clinical and Experimental Medicine, University of Pisa, Pisa, Italy; ^2^ Department of Surgical, Medical and Molecular Pathology and Critical Care Medicine, University of Pisa, Pisa, Italy; ^3^ Division of General and Transplant Surgery, Azienda Ospedaliero-Universitaria Pisana, University of Pisa, Pisa, Italy; ^4^ General Surgery Unit, Department of Surgery, Translational and New Technologies in Medicine, University of Pisa, Pisa, Italy

**Keywords:** IgG4-related disease, CXCL12, CXCR4, fibrosis, inflammation, NETs (neutrophil extracellular traps)

## Abstract

**Background:** SDF-1/CXCL12 is a chemokine with pleiotropic functions in hematopoietic stem cell niche homeostasis, germinal center architecture, B cell maturation, neoangiogenesis, and fibrosis. Recently, the CXCL12/CXCR4/CXCR7 axis was associated with cancer metastasis and autoimmune diseases. The IgG4-related disease (IgG4-RD) is a pathological condition characterized by IgG4+ plasma cells infiltrating fibrotic lesions. The aim of this research is to investigate the relevance of SDF-1/CXCL12 in IgG4-RD.

**Materials and Methods:** Peripheral blood samples were collected before therapy from a single-center cohort of 28 IgG4-RD patients, fulfilling the ACR-EULAR classification criteria. Clinical and serological data were obtained for each patient. In total, 14 healthy donors (NHS), 9 patients with pancreatic ductal adenocarcinoma (PDAC), and 9 with Sjogren syndrome (SSj) were recruited as controls and screened for circulating SDF-1/CXCL12 by ELISA. Moreover, paraffin-embedded pancreatic biopsies obtained from patients with IgG4-RD (*n* = 7), non-autoimmune pancreatitis (*n* = 3), PDAC (*n* = 5), and control tissues (*n* = 4) were analyzed to study the tissue expression and localization of SDF-1/CXCL12 and one of its receptors, CXCR4, and their potential relation with neutrophil extracellular traps (NETs).

**Results:** IgG4-RD patients had higher serum levels of SDF-1/CXCL12 than normal controls (*p* = 0.0137). Cytokine levels did not differ between the IgG4-RD autoimmune pancreatitis (AIP) and retroperitoneal fibrosis nor between the single- and multiple-organ involvement. No correlation was seen with the IgG4-RD Responder Index, IgG4 levels, white blood cells, or inflammatory markers in the serum. When compared to SSj, the IgG4-RD AIP subgroup presents higher amounts of serum SDF-1/CXCL12 (*p* = 0.0275), while no differences are seen in comparison with PDAC. The expression of SDF-1/CXCL12 in the tissue was significantly higher in the IgG4-RD tissue than the normal pancreas, and the tissue with the high SDF-1/CXCL12 expression is characterized by the overall inflammatory cell infiltration, fibrosis, and high level of NETs.

**Conclusion:** Modulating B cell development, neoangiogenesis and fibrosis, and SDF-1/CXCL12 may play a role in IgG4-RD. The higher levels observed in IgG4-RD, as compared to SSj, which closely mimics the disease, can be related to a different pattern of lesions, with prevalent fibrosis seen in IgG4-RD. Taken together, these findings suggest that drugs acting on the CXCL12/CXCR4/CXCR7 axis may affect IgG4-RD.

## Introduction

IgG4-related disease (IgG4-RD) is a rare condition characterized by fibro-inflammatory lesions in one or many organs with peculiar histological features, such as tissue fibrosis with a storiform pattern, a diffuse lymphoplasmacytic infiltrate, obliterative phlebitis, mild to moderate eosinophil infiltrate, and abundance of IgG4+ plasma cells (Deshpande et al., 2012; Stone et al., 2012; Inoue et al., 2015).

Oligoclonal somatically hypermutated plasmablasts that produce IgG4 are detected in the peripheral blood (Mattoo et al., 2014; Doorenspleet et al., 2016). This population of B cells may be considered not only as a biomarker of the disease but also as a disease activity marker. In fact, plasmablasts are reduced during disease remission and reemerge during relapse (Wallace et al., 2015; Lin et al., 2017).

Little is known about B cell maturation and mutation in IgG4-RD, but tertiary lymphoid structures have been described in affected organs (Deshpande et al., 2012) and, as demonstrated in other disorders (Corsiero et al., 2016a), may represent sites of pathogenic B cell expansion.

The disorder can affect almost any organ: the pancreas, retroperitoneum, lymph nodes, salivary glands, and kidneys are the most frequently involved ones (Mahajan et al., 2014; Puxeddu et al., 2018a; Capecchi et al., 2021).

Diagnosis of IgG4-RD is based on a set of clinical, serological, and pathological criteria (Wallace et al., 2020), and the histological picture is critical for diagnosis.

The hallmark of IgG4-RD is represented by a typical pattern of fibrosis, rarely detected in other inflammatory disorders, with spindle cells (fibroblasts and myofibroblasts) radiating from a center (Deshpande et al., 2012). As in any fibrotic disorder, myofibroblasts, the main cells involved, exert their profibrotic activity deposing excessive extracellular matrix components, and the proportion of these cells decrease in advanced stages of the disease, characterized by a relatively acellular tissue.

Presently, few data are available on the circuits controlling the processes leading to tissue fibrosis in IgG4-RD. It has been suggested that a critical step in inducing the typical lesions is the release of profibrotic cytokines, but so far this hypothesis has only been partially investigated (Della-Torre et al., 2015; Puxeddu et al., 2018b; Capecchi et al., 2018).

Recent data draw attention to the role of SDF-1/CXCL12 as a mediator involved in recruiting circulating fibrocytes to the inflamed tissue in chronic periaortitis, a fibrotic disorder in the spectrum of IgG4-RD (Nicastro et al., 2019).

SDF-1/CXCL12 is a chemokine originally identified as a product of murine bone marrow stromal cells that interacts with two receptors, CXCR4 and CXCR7 (Karin, 2010). In embryonic life, this chemokine controls the proliferation and differentiation of immature progenitor cells (Nagasawa et al., 1996). In adults, SDF-1/CXCL12, constitutively expressed in bone marrow, the skin, the heart, and brain endothelium, regulates immature and maturing leukocyte trafficking to these tissues (Dar et al., 2006).

SDF-1/CXCL12 is also a potent chemoattractant for bone marrow eosinophils (Wong and Jelinek, 2013) and, in inflammatory processes, mediates leukocyte entry to inflammatory sites.

The pleiotropic effects of this chemokine suggest its possible involvement in multiple aspects of IgG4-RD.

In this study, we thus analyzed the serum levels of SDF-1/CXCL12 in IgG4-RD patients, focusing in particular on the subgroup with pancreatic involvement. The pancreatic expression of SDF-1/CXCL12 and its receptor CXCR4 was also studied in a subset of IgG4-RD patients with autoimmune pancreatitis (IgG4-RD AIP) and compared to what was observed in the pancreatic ductal adenocarcinoma (PDAC). In addition, the SDF-1/CXCL12 expression in the pancreatic tissue was evaluated in relation to the inflammatory infiltrate, particularly neutrophils, and neutrophil extracellular traps (NETs).

## Materials and Methods

### Patients and Controls

In total, 28 patients fulfilling the criteria for the diagnosis of IgG4-RD were included in the study (Wallace et al., 2020). Here, 14 sex- and age-matched normal healthy subjects (NHS) were used as controls, and 8 patients affected by Sjogren’s syndrome (SSj) (diagnosed according to ACR criteria) (Shiboski et al., 2017) and 9 affected by PDAC were also studied as disease controls. The IgG4-Related Disease Responder Index (IgG4-RD RI) was calculated as described by Carruthers et al. (2012). The study was approved by the local ethics committee (protocol 3661/2012), and patients gave their written informed consent.

### Detection of Soluble Mediators

The levels of SDF-1/CXCL12 in the sera of IgG4-RD patients and controls were measured by ELISA (Human SDF-1/CXCL12 Quantikine, R&D Systems, Inc., Minneapolis, MN, USA), according to the manufacturer’s instructions.

### Tissue Biopsies and H&E Staining

Paraffin-embedded pancreas biopsies from seven IgG4-RD patients, three not-autoimmune pancreatitis, five PDAC, and four normal tissues (adjacent to the tumor) were obtained from the Pathology Unit, Department of Surgical Pathology, University of Pisa. In order to confirm the original diagnosis, all the tissue sections were reviewed by an expert pathologist. All the biopsies used in this study were fixed in a 10% buffered formalin solution, paraffin embedded, and cut at 3 μm. H&E staining was performed on all samples. Each sample was analyzed by an expert pathologist and scored from 0 to 3 for the level of fibrosis, follicles, lymphocytes, neutrophils, eosinophils, and plasma cells.

### Immunofluorescence Staining for CXCR4, SDF-1/CXCL12, and NETs

Deparaffinized pancreatic biopsies underwent antigen retrieval at 95°C with a low pH buffer solution followed by block of not-specific binding. The sections were then incubated with the proper combination of primary antibodies; for CXCR4 and SDF-1/CXCL12, the slides were incubated overnight at 4°C with a goat anti-mouse IgG1 antibody specific for SDF-1/CXCL12 (R&D), and after PBS washing, the sections were incubated for 1 h at RT with a goat anti-mouse IgG2b antibody specific for CXCR4 (R&D). For detection of NETs, the slides were incubated for 1 h at RT with a goat polyclonal anti-rabbit antibody specific for MPO (Dako) and a goat anti-chicken antibody specific for H2B (Abcam).

After washing, the sections were incubated with the appropriate combination of fluorochrome-conjugated secondary antibodies. Alexa 488–conjugated goat anti-mouse IgG1 and Alexa 555–conjugated goat anti-mouse IgG2b (Invitrogen) were used for SDF-1/CXCL12 and CXCR4, respectively. Alexa 488–conjugated goat anti-rabbit and Alexa 555–conjugated goat anti-chicken (Invitrogen) were used for MPO and H2B, respectively.

Cell nuclei were counterstained with DAPI. Negative controls included omission of the primary antibody followed by the proper secondary antibody.

All the sections were visualized using an Olympus BX-41 microscope. Stained cells were counted in five 40X microscopic fields per section.

### Analysis of Immunofluorescence Staining

The presence of SDF-1/CXCL12 and its receptor CXCR4 was evaluated by a semi-quantitative score. In detail, in each tissue slide, both CXCR4 and SDF-1/CXCL12 were scored from 0 (no cells) to 3 (high number of cells). A median value was obtained for the IgG4-RD sample tissues and control groups (not-autoimmune pancreatitis, PDAC, and normal tissue). Both SDF-1/CXCL12 and CXCR4 expression levels were evaluated in relation to the level of fibrosis and the presence of follicles, lymphocytes, plasma cells, eosinophils, and neutrophils, previously evaluated by H&E staining. Finally, in the same tissue, both CXCR4 and SDF-1/CXCL12 expressions were correlated with NETs (evaluated using immunofluorescence staining by the co-expression of MPO and H2B in five 40X microscopic fields per section). The levels of SDF-1/CXCL12 were considered low with a score of 0 or 1 and high with a score of 2 or 3.

### Statistical Analysis

A non-parametric Mann–Whitney *U*-test was used to compare the patients’ group and the normal subjects. *p* < 0.05 was considered statistically significant.

## Results

### Patient Cohort

The clinical and serological features of the IgG4-RD patients are summarized in Table 1.

**TABLE 1:** Demographic and serological characteristics of IgG4-RD patients.

	IgG4-RD patients (*n =* 28)
Age (years) (mean and range)	62 (39-81)
Sex (M/F)	17/11
Disease duration (months) (mean and range)	23 (0-413)
IgG (mg/dl) median (IQR)	1342 (1111–1833)
IgG4 (mg/dl) median (IQR)	210 (99.5–218)
IgG4/IgG (%) median (IQR)	17.57 (11.78–23.16)
IgE (mg/dl) median (IQR)	57 (15.5–98)
ESR (mm/h) median (IQR)	25 (10–70)
CRP (mg/L) median (IQR)	0.4 (0.16–1.13)
Eosinophils (n°/µl) median (IQR)	200 (87.5–282.5)
IgG4-RD RI median (IQR)	7.5 (5–10)

IQR, interquartile range; *ESR*, erythrocyte sedimentation rate; *CRP*, C-reactive protein; *IgG4-RD RI*, IgG4-related disease Responder Index.

In total, 13 patients had a single-organ involvement, while 8 patients had a two-organ involvement, and 7 had multi-organ diseases (three or more organs affected). The most frequent organs involved were pancreas (*n* = 13); retroperitoneum (*n* = 9); lymph nodes (*n* = 8); salivary glands (*n* = 7); orbits and lacrimal glands (*n* = 4); the kidney, liver, and thyroid (*n* = 3); and lungs, large vessels, and the pituitary gland (*n* = 2). Skin involvement was observed only in one patient. The median of the IgG4-RD Responder Index was 7.5, indicating an active disease in most of the patients. The median of circulating eosinophils was 200/μl (87.5–282.5 IQR) with a value > 700/μl in 2 out of 28 patients. Three patients were previously treated with immunosuppressant (rituximab or cyclophosphamide), while eight patients were under treatment with low-dose steroids (mean dose 8 mg/die prednisone equivalent, SD ± 11).

### Increased SDF-1/CXCL12 Serum Levels in IgG4-RD

**FIGURE 1:**
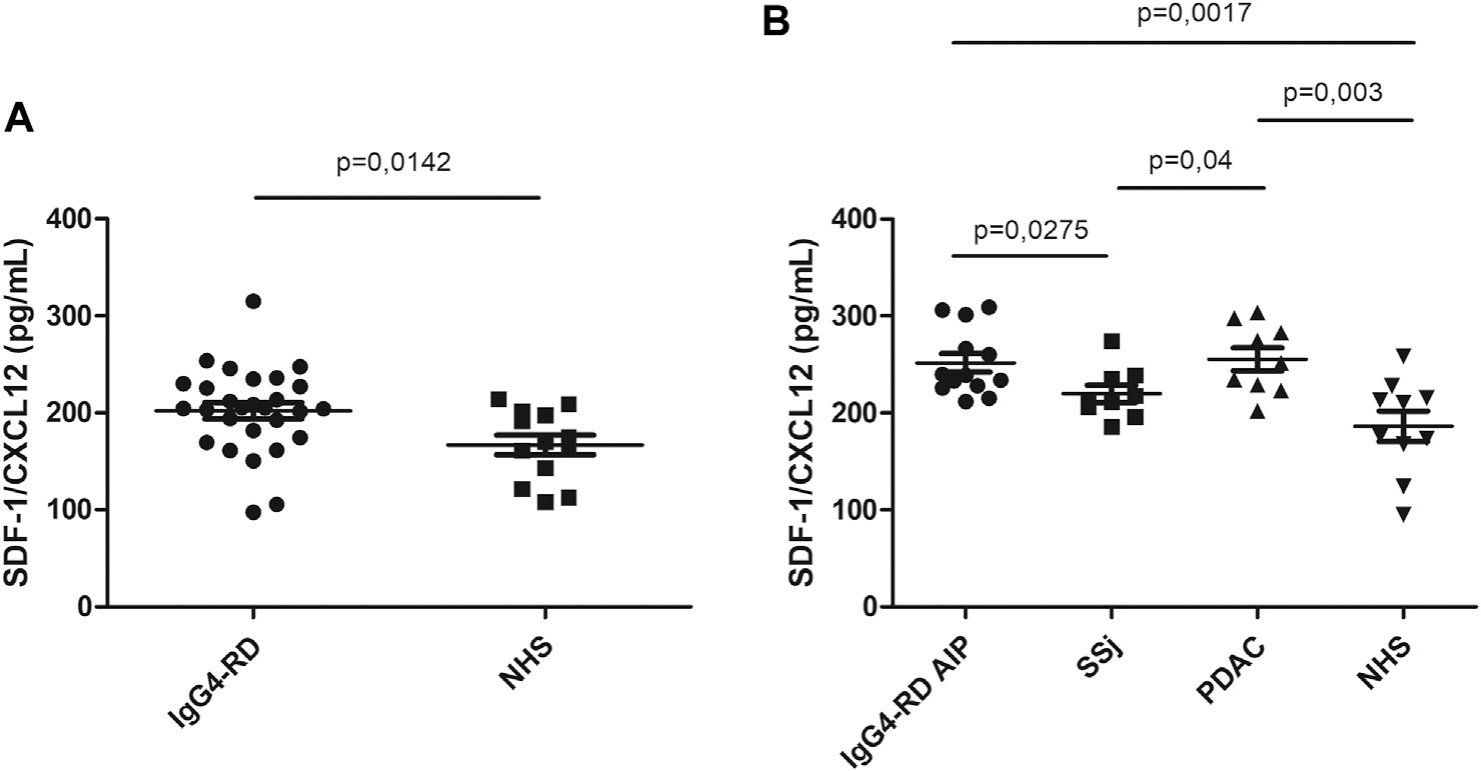
SDF-1/CXCL12 serum level in IgG4-RD patients and healthy controls (NHS) **(A)**. SDF-1/CXCL12 serum level in IgG4-RD autoimmune pancreatitis (AIP), Sjogren syndrome (SSj), pancreatic adenocarcinoma (PDAC), and healthy controls (NHS) **(B)**.

SDF-1/CXCL12 serum levels were significantly higher in IgG4-RD patients compared to NHS (*p* = 0.0142) (Figure 1A). The subgroup of IgG4-RD AIP was compared with SSj and PDAC patients. SDF-1/CXCL12 levels were higher in IgG4-RD AIP than in SSj patients but comparable to those observed in PDAC (Figure 1B).

In IgG4-RD patients, the levels of SDF-1/CXCL12 were not related to inflammatory markers (erythrocyte sedimentation rate or C-reactive protein or disease activity evaluated by the Responder Index (data not shown)). Although in the literature it has been demonstrated that SDF-1/CXCL12 is an eosinophil chemoattractant (Nagase et al., 2001), we did not found any correlation between the serum level of this cytokine and the number of circulating eosinophils.

### Increased SDF-1/CXCL12 Expression in IgG4-RD Tissue

**FIGURE 2:**
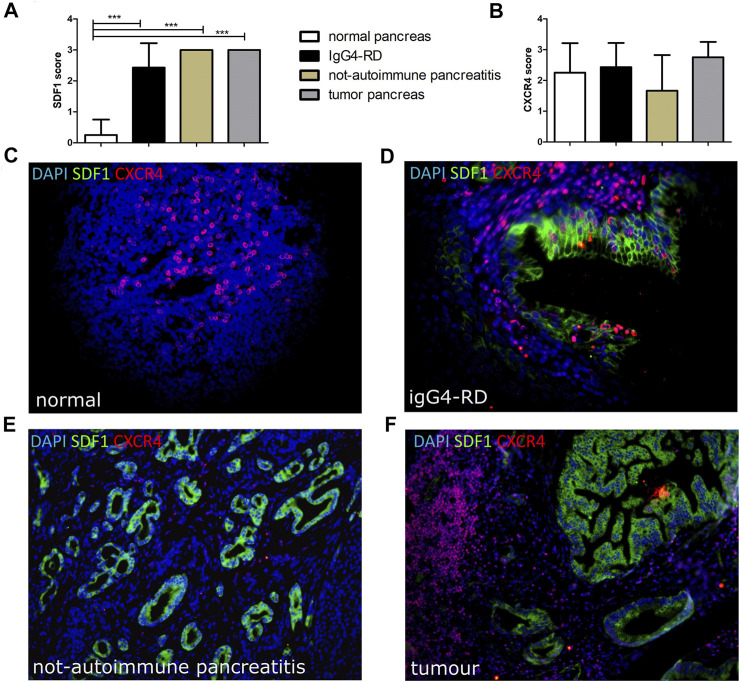
SDF-1/CXCL12 **(A)** and CXCR4 **(B)** score in pancreatic tissues from normal, IgG4-RD, not-autoimmune pancreatitis, and tumor pancreas. A representative image of a double staining SDF-1/CXCR4 in normal pancreas **(C)**, IgG4-RD pancreas **(D)**, not-autoimmune pancreatitis **(E)**, and tumor pancreas **(F)** is shown. ∗∗∗ = *p* < 0.001.

The SDF-1/CXCL12 expression was significantly higher in IgG4-RD with respect to normal pancreas tissue, comparable to those found in different disease groups including PDAC and in not-autoimmune pancreatitis (Figure 2A). Conversely, we did not find any significant difference in the CXCR4 level of expression and localization among the different disease groups (Figure 2B). SDF-1/CXCL12 was mainly expressed in ducts and acini whereas CXCR4 was very rarely expressed in SDF-1/CXCL12-positive cells (Figures 2C–F).

### SDF-1/CXCL12 Expression and the Overall Inflammatory Cell Infiltration and Fibrosis

**FIGURE 3:**
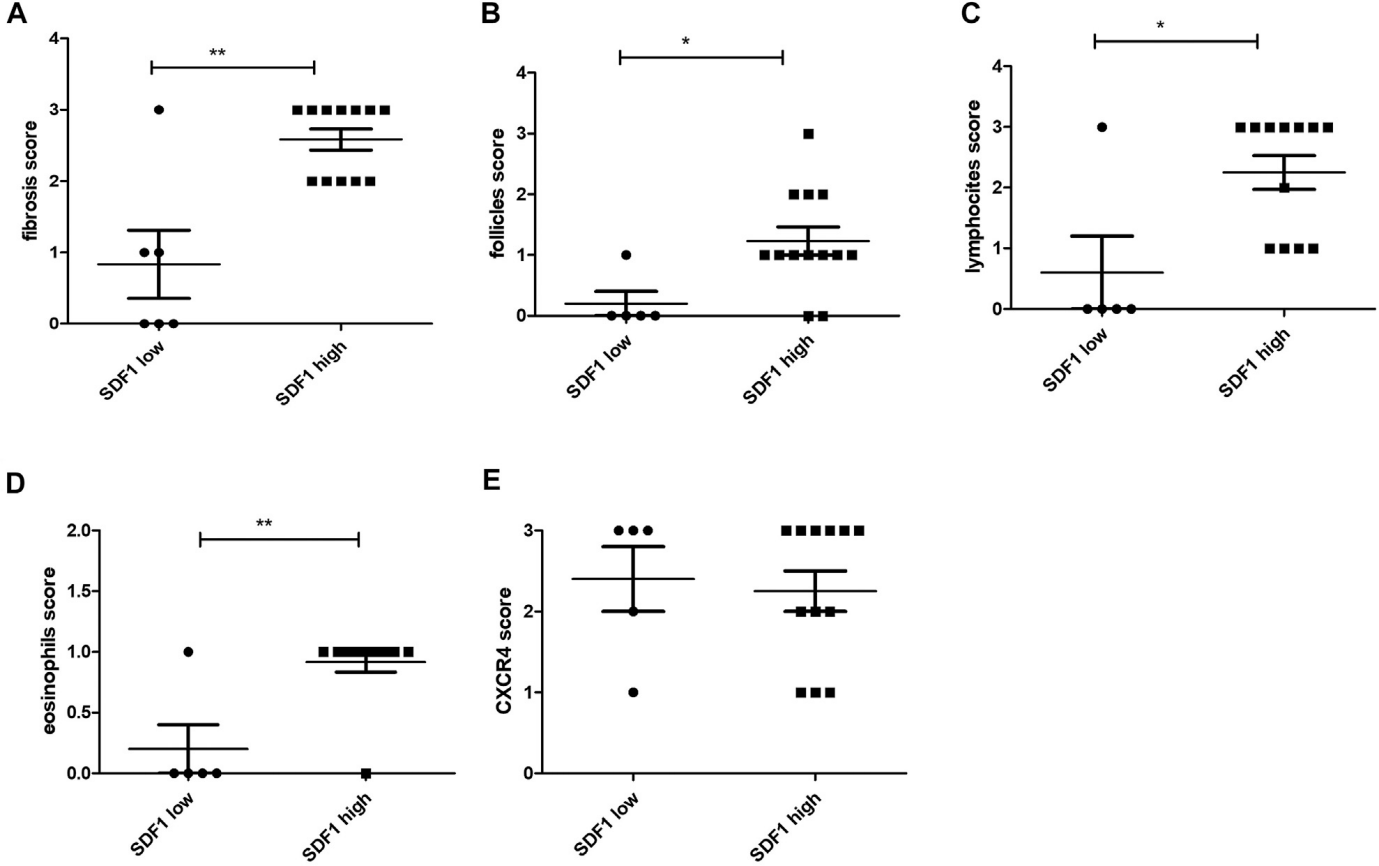
SDF-1/CXCL12 expression in the pancreas tissue related to the fibrosis score **(A)**, the follicle score **(B)**, the lymphocyte score **(C)**, the eosinophil score **(D)**, and the CXCR4 score **(E)**. ∗ = *p* < 0.05; ∗∗ = *p* < 0.01.

In the pancreatic tissues with high expression of SDF-1/CXCL12, significantly higher levels of fibrosis (Figure 3A), follicles (Figure 3B), lymphocytes (Figure 3C), and eosinophils (Figure 3D) were also found. On the contrary, no differences between high and low expression of SDF-1/CXCL12 were observed stratifying for the CXCR4 expression (Figure 3E). Similarly, no differences between the CXCR4 expression and the level of fibrosis, the number of follicles, lymphocytes, neutrophils, eosinophils, or plasma cells were detected (data not shown).

### An Aberrant Level of NETs Is Characteristic of Tissue Expressing High Levels of SDF-1/CXCL12

**FIGURE 4:**
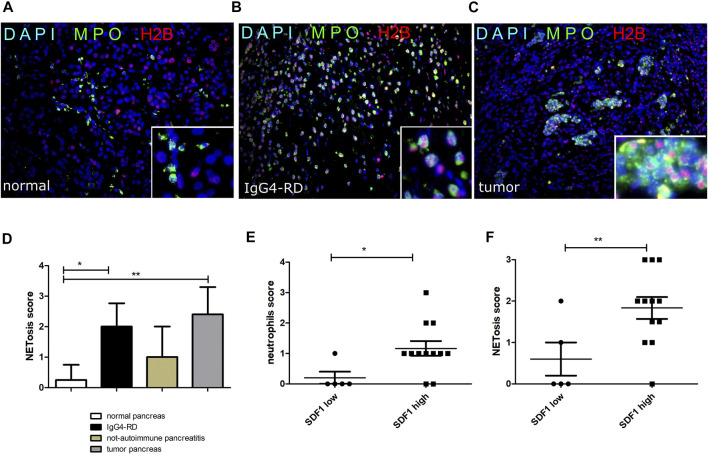
NETs (double H2B and MPO staining) in normal pancreatic tissue **(A)**, IgG4-RD pancreas **(B)**, and tumor pancreas **(C)**. A semi-quantitative analysis of NETosis in the different groups is reported **(D)**. The neutrophil **(E)** and NETosis **(F)** score in pancreas reflects the SDF-1/CXCL12 expression in pancreatic tissue. ∗ = *p* < 0.05; ∗∗ = *p* < 0.01.

As reported in Figures 4A-C, NETs were more frequent in pancreatic tissues from IgG4-RD and PDAC compared to the control. Semi-quantitative analysis of the NET expression revealed a significantly increased level of NETs in IgG4-RD and PDAC pancreatic tissues compared to normal control (*p* < 0.05 and *p* < 0.01, respectively) (Figure 4D). By analyzing the data from all the pancreatic tissue biopsies, we observed in the pancreatic tissues with high expression of SDF-1/CXCL12 a significantly higher level of neutrophils (Figure 4E) and NETs (Figure 4F) than in the tissues with low SDF-1/CXCL12 expression (*p* < 0.05 and *p* < 0.01, respectively).

## Discussion

In this study, we analyzed the serum levels of SDF-1/CXCL12 in IgG4-RD patients and the pancreatic expression of the chemokine in the subgroup of IgG4-RD with autoimmune pancreatitis.

The results indicate that chemokine is overexpressed in the sera of IgG4-RD at levels comparable to what was observed in PDAC patients. SDF-1/CXCL12 serum levels are not correlated with the disease activity index or with acute phase reactants and, thus, cannot be considered a biomarker of disease exacerbations. Patients affected by chronic periaortitis, a fibro-inflammatory condition in the spectrum of IgG4-RD, show a similar increase in SDF-1/CXCL12 serum levels (Puxeddu et al., 2018a). Only a subset of chronic periaortitis patients, however, can be diagnosed as IgG4-RD. According to our findings, SDF-1/CXCL12 is elevated in all IgG4-RD patients, especially in those with pancreatic involvement. Under this respect, the overexpression of the chemokine in the pancreas from AIP, not-autoimmune pancreatitis, and PDAC is of interest as compared to normal tissue. In all these conditions, the SDF-1/CXCL12 expression is correlated with overall fibrosis, suggesting a role of this chemokine in fibrotic tissue remodeling in all these disorders. SDF-1/CXCL12, locally produced mainly by fibroblasts and hepatic stellate cells, exerts profibrotic activity by multiple mechanisms. Activation and proliferation of fibroblasts with increased production of extractable matrix components have been described as the effect of the chemokine (Jackson et al., 2017). A second mechanism is tissue recruitment of circulating fibrocytes and mesenchymal progenitor cells (CD45^+^, collagen-1+) that migrate to sites of injury and play an important role in disorders characterized by excessive collagen deposition (Herzog and Bucala, 2010). Fibrocytes produce a number of extracellular matrix proteins, including collagen-1, collagen-3, and fibronectin (Ashley et al., 2017). In a mouse model of lung fibrosis, it has been shown that human fibrocytes migrate in response to SDF-1/CXCL12 and localize to lungs if injected in bleomycin-treated animals (Phillips et al., 2004). An increase in the number of collagen-positive fibrocytes was detected in chronic periaortitis patients, both in peripheral blood and in the affected tissue (Nicastro et al., 2019). Similarly, in IgG4-RD patients, SDF-1/CXCL12 may recruit fibrocytes to affected organs, driving tissue fibrosis.

In PDAC, overexpression of SDF-1/CXCL12 has often been reported, at least in part, mediated by the production of the hypoxic inducible factor (HIF) in the hypoxic tumor environment. The profibrotic activity of the chemokine supports tumor growth, protecting cancer cells from the host’s immune system and creating niches for metastatic growth (Righetti et al., 2019).

In the tissue sections we studied, the SDF-1/CXCL12 expression reflects the number of lymphoid follicles, and eosinophil and neutrophil infiltrate.

SDF-1/CXCL12 plays an important role in B cell homing and differentiation in germinal centers. Ectopic germinal centers have been described in salivary glands of IgG4-RD patients (Maehara et al., 2012), and the role of SDF-1/CXCL12 in the formation of these ectopic lymphoid structures are well established (Corsiero et al., 2012).

Thus, it is not surprising that in different disorders, the SDF-1/CXCL12 expression is related to the number of lymphoid follicles.

The relation with eosinophil and neutrophil infiltrates suggests the role of SDF-1/CXCL12 in mediating the migration of these cells into pancreatic tissue. SDF-1/CXCL12 is by itself a weak neutrophil chemoattractant but synergizing with other chemokines like IL-8 may increase neutrophil infiltration (Janssens et al., 2018).

SDF-1/CXCL12 signaling is mediated by the interaction with CXCR4, a G-coupled receptor that is activated also by ubiquitin, and by the binding of a macrophage migration inhibitory factor. CXCR4 is diffusely expressed in the pancreas, indicating that the chemokine may exert its activities in the tissue through receptor binding. No differences in CXCR4 distribution or density were detected in different diseases we studied; thus, chemokine and the receptor expression are not parallel and in fact has been reported that the CXCR4/SDF-1 system is regulated at the level of the receptor expression (Nagase et al., 2001). However, these data do not allow drawing firm conclusions on the functional activity of SDF-1/CXCL12 in the pancreas.

In fact, splice variants of SDF-1/CXCL12 give rise to different isoforms with a tissue-specific pattern of expression: CXCL12δ, CXCL12ε, and CXCL12ϕ are most abundantly expressed in the pancreas. The isoforms share the first 67 amino acids but differ in length and in signaling efficiency. Only splice variant-specific antibodies may shed light on the pattern of the isoform expression in the tissue. Moreover, the biological activity is regulated by posttranslational modification of the chemokine, such as citrullination and tyrosine nitration (Struyf et al., 2009; Janssens et al., 2016).

SDF-1/CXCL12 may also contribute to organ damage, mediating neutrophil infiltration of the pancreas.

In IgG4-RD and PDAC, neutrophils infiltrating the pancreas frequently undergo NETosis, a form of programmed death that represents a critical defense mechanism in innate immunity but also a potential mechanism of tissue damage. By the extrusion of chromatin fibers coated with granule enzymes and other cytoplasmic constituents, neutrophils entrap the microorganisms too big to be phagocytosed (Brinkmann et al., 2004). NETs, however, can be directly harmful to the surrounding cells, can lead to the production of ROS and other inflammatory mediators, and may also represent a molecular platform for autoantibody induction (Corsiero et al., 2016b).

In PDAC, IL-17 mediates neutrophil recruitment and induces NETosis. NETs enhance migration and activate pancreatic stellate cells that form dense stroma and enable tumor proliferation (Miller-Ocuin et al., 2019).

One of the NET constituents is the antimicrobial peptide cathelicidin, which forms complexes with RNA; complexed RNA is protected from degradation and transported into the endosomal compartment of dendritic cells where it stimulates the production of IFN alpha (Ganguly et al., 2009). Moreover, activation of dendritic cells is triggered by RNA-cathelicidin complexes. A dense plasmacytoid dendritic cell infiltrate characterizes the affected pancreas in AIP. As reported by Arai et al., in IgG4-RD patients, NET-stimulated dendritic cells release IFN alpha and induce the production of IgG4 in cocultured B cells (Arai et al., 2015). Thus, in IgG4-RD and PDAC, SDF-1/CXCL12 may affect inflammatory cell migration and B cell homing in the pancreas. The stimuli responsible for NET formation may differ in the two disorders, and the contribution of NETosis to disease progression in PDAC and IgG4 production should be further explored.

## Data Availability

The raw data supporting the conclusion of this article will be made available by the authors, without undue reservation.
